# IMPARTER, Phase 1 of an intervention to improve patients’ understanding of gene expression profiling tests in breast cancer

**DOI:** 10.1007/s10549-021-06491-2

**Published:** 2022-01-04

**Authors:** L. J. Fallowfield, D. Farewell, H. Jones, S. May, S. Catt, R. Starkings, V. Jenkins

**Affiliations:** 1 Sussex Health Outcomes Research & Education in Cancer, Brighton & Sussex Medical School, University of Sussex, Brighton, UK; 2 Division of Population Medicine, School of Medicine, Cardiff University, Cardiff, UK

**Keywords:** Patient information, Gene expression profiling tests, Early breast cancer

## Abstract

**Purpose:**

To compare participants’ knowledge about gene expression profiling (GEP) tests and recurrence risks after reading an information leaflet with that following viewing of an information film.

**Methods:**

Using a randomised cross-over design, at time-point one (T1), women aged 45–75 years without breast cancer either read leaflets or watched information films about Onco*type* DX or Prosigna tests. Participants answered nine questions assessing knowledge (maximum score 18). Next-day information in the opposite modality was provided and knowledge re-assessed. Additional questions probed which format was easiest to understand, participants’ preferences for film or leaflet and their reasons for these.

**Results:**

120 women participated (60 received Onco*type*DX films and leaflets; 60 received the Prosigna versions). T1 mean knowledge scores were higher following film viewing (13.37) compared with that after reading leaflets (9.25) (mean difference 4.1; *p* < 0.0001; 95% CI 3.2, 5.0). When participants read leaflets first and subsequently viewed films, all increased their scores (mean + 6.08, from T1 of 9.25, *p* < 0.0001; 95% CI 5.44, 6.72). When films were viewed first, followed by leaflets, (36/60, 60%), participants’ scores declined (mean-1.55 from T1 of 13.37, *p* < 0.001; 95% CI -2.32, -0.78). A majority of participants expressed preferences for the films (88/120, 73.3%) irrespective as to whether they described Onco*type*DX or Prosigna. Reasons included the clarity, ease of understanding, visual material and reassuring voice-over.

**Conclusion:**

Discussions between oncologists and patients about recurrence risk results can be challenging. Information leaflets may aid understanding but often employ complex language. Information films significantly improved knowledge and were preferred by participants.

## Introduction

Advances made in our understanding of the molecular biology of breast cancer have undoubtedly improved diagnostic testing and increased the therapeutic options available to patients. Importantly, results from genomic testing permit more accurate tailoring of treatment, allowing more women potentially to live for longer with a better quality of life. In early breast cancer, adjuvant chemotherapy can now be offered only to those who are at highest risk of recurrence, sparing low-risk patients the side effects of cytotoxic treatment. Onco*type*DX and Prosigna are two gene expression profiling (GEP) tests that provide recurrence risk results; these help determine the likely therapeutic benefit of adding chemotherapy to hormones alone in patients with early, oestrogen-positive, HER2-negative breast cancer [1, 2].

Educational workshops—talking about risk in the context of gene expression profiling tests (TARGET), revealed the complexity of conversations between health care professionals (HCPs) and patients about recurrence risks and optimal treatments [3]. Medicine is not an exact science, so discussing risks and uncertainty is challenging especially when communicating with anxious patients with low tolerances of uncertainty, who may feel that more treatment must be better than less. Such problems increase if GEP scores are intermediate or close to either high- or low-risk of recurrence thresholds.

Many clinical teams provide their patients with information leaflets to aid understanding about GEP tests, recurrence scores and implications for treatment; these are often written by academic or commercial sponsors and designed to meet certain ethical and regulatory guidelines which might not educate the end-user effectively. Although HCPs and experienced patient advocates can provide valuable insights with the review and writing of information leaflets, they may all be inured to some of the jargon and complexities used. Worldwide health literacy and numeracy are poorer than realised; for example, 42% of UK working age adults are unable to understand everyday health materials, a figure that rises to 61% if numeracy skills are also required for comprehension [4]. Even highly educated individuals have difficulty with understanding simple probabilities and basic numeracy tasks [5].

When put through a standard readability scale [6], excerpts from leaflets explaining the purpose of Onco*type*DX and Prosigna were judged as ‘very’ or ‘fairly’ difficult requiring the reading ages of college graduates or 12th-grade students, respectively. Importantly, these scales only assess readability not comprehension, although both are linked. An inability to read something easily likely impacts upon understanding, which in itself is crucial for informed decision-making.

One study showed breast cancer patients had low knowledge about Onco*type*DX recurrence risk with > 50% unable to answer questions correctly despite having received the answers minutes before. Patients with lower health literacy had poorer knowledge than those with higher health literacy [7]. Another study showed the association of health literacy with greater retention of information about GEP test results and preferences for active decision-making. Authors also reported that even patients with lower health literacy still desired information about their recurrence risk results [8].

Anyone preparing patient information must appreciate that individuals not only have varied literacy and numeracy skills, but that attitudes and personality dispositions may also influence interpretation of, and reactions to, risk information. Some may desire actual risk estimates, while others become confused by these, preferring to base decisions on other types of information or combinations of verbal descriptors with numbers.

Clearly different ways of presenting patient information about risk is required to augment face-to-face communication and ensure consistent messaging that enhances educated decision-making. In particular, there is evidence that user-friendly information films might improve patient knowledge and understanding and be more accessible to certain individuals [9]. For example, a study comparing video with print materials showed that video presentation was advantageous, especially to participants with lower literacy skills [10].

We developed two eight-minute films explaining what GEP tests are, why they are used in breast cancer and how results help determine whether or not chemotherapy is recommended as a treatment option. Both films were conversational in style, with a generic introduction incorporating simple explanatory graphics and visual material of doctors and patients taken from the TARGET educational materials [3]. Following this introduction, separate sections explained either the Onco*type* DX or Prosigna tests and recurrence risk results. Ten lay volunteers viewed draft versions, then answered questions about key points and provided general feedback. Their comments highlighted areas of potential misunderstanding within the graphics or script voice-over. Final versions were produced and checked ensuring that information covered was compatible with that found in the leaflets.

## Methods and statistics

### Recruitment

Due to the SARS‑CoV‑2 pandemic, we were unable to recruit patients from clinics, so used a study design that avoided face-to-face contact with participants. Snowballing and then purposive sampling were employed to recruit 120 women from a varied socio-educational distribution and of appropriate ages to mirror those of women with breast cancer. Participants had to be able to read and speak English and have access to a computer, tablet, or smartphone. We excluded women with a current or previous cancer. All interviews were conducted remotely on-line using Zoom or by telephone as preferred.

### Design

**Fig. 1:**
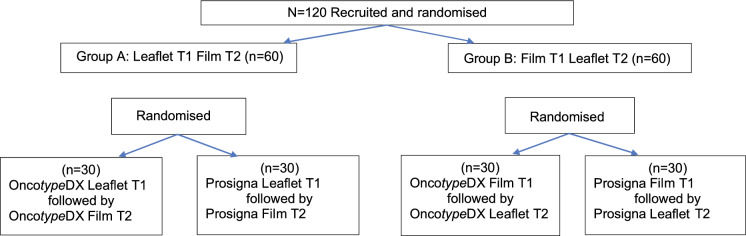
Study design

A randomised factorial cross-over study was employed (see Fig. 1). Participants were allocated to: Group A who read an Onco*type* DX or Prosigna leaflet before their first assessment (T1) and then viewed the corresponding film at the second (T2) assessment, or Group B who viewed a Onco*type* DX or Prosigna film first at T1, followed by reading the leaflet at T2. Allocation was stratified ensuring that groups had participants of similar ages and socio–educational characteristics. Brighton and Sussex Medical School Research Governance Ethics Committee granted approval for the study (ER/LESLEY/1). We audio-recorded participants’ verbal consent.

Assessments comprised study-specific interview schedules that measured participants’ knowledge and understanding of nine key information facts found in both the films and leaflets. The items probed included the nature and purpose of GEP testing and the meaning of different recurrence risk results. The maximum possible score was 18, with higher scores indicating better understanding. At T2, additional questions explored: participants’ preferences for either the film or leaflet, their perceptions as to which had most aided their understanding and reasons for these views. Participants could read or watch the information as many times as they liked on whatever device they preferred—laptop, tablet or smart phone. They were encouraged to make notes, which they could refer to when answering questions.

### Primary outcome

The primary outcome was the comparison at T1 of participants’ knowledge scores after reading GEP information leaflets or viewing the information films.

### Secondary outcomes

Secondary outcomes were as follows: (1) the influence that order of imparting information might have on knowledge scores (2) participants’ perceptions as to which modality most helped their understanding, (3) overall preferences for either film or leaflet and (4) reasons for perceptions and preferences.

### Statistical methods

The data were analysed with R version 4.0.3 software and the tidyverse and PropCIs packages [11, 12]. T-tests were used to compare means of knowledge scores; these were unpaired when contrasting groups, but paired when comparing the same individuals between time-points. Paired *t*-tests of changes used Welch’s approximation [13]. For tests of single proportions or a comparison of two proportions, confidence intervals were calculated using Newcombe’s method for Wilson’s procedure [14, 15].

## Results

We recruited 120 participants over 9 weeks. (Table 1 shows socio-demographic characteristics.) Many knew close friends (49/120; 40.8%) or family members (27/120; 22.5%) who had received chemotherapy. Participants mainly viewed leaflets and films on a tablet (68/120) or laptop (69/120), and most read leaflets or viewed films approximately twice [2.54 (SD 1.40); 2.38 (SD 0.97)], respectively.

**Table 1:** Participants’ characteristics

	All *n* = 120 (%)	Group A Onco*type* DX *n* = 60	Group B Prosigna *n* = 60
*Age range (years)*			
45–55	33 (27.5)	19 (31.7)	14 (23.3)
56–65	61 (50.8)	32 (53.3)	29 (48.3)
66–70	17 (14.2)	5 (8.3)	12 (20)
71–75	9 (7.5)	4 (6.7)	5 (8.3)
*Formal education*			
O levels/GCSEs equivalent	23 (19.2)	8 (13.3)	15 (25)
Trade/tech/vocational	12 (10)	6 (10)	6 (10)
A levels/Scottish highers	18 (15)	10 (16.7)	8 (13.3)
Teacher training	9 (7.5)	4 (6.7)	5 (8.3)
University degree	33 (27.5)	19 (31.7)	14 (23.3)
Post-grade/professional	25 (20.8)	13 (21.7)	12 (20)
*Employment status*			
Full time	30 (25)	17 (28.3)	13 (21.7)
Part time	21 (17.5)	15 (25)	6 (10)
Self-employed	9 (7.5)	2 (3.3)	7 (11.7)
Retired	53 (44.2)	23 (38.3)	30 (50)
Unemployed	3 (2.5)	1 (1.7)	2 (3.3)
Look after the home	4 (3.3)	2 (3.3)	2 (3.3)

### Primary outcome

At T1, there was a significant difference in knowledge scores of 4.12 [*p* < 0.0001, 95% CI (3.2, 5.0)]. Participants viewing the film had a mean knowledge score of 13.37 (IQR 12.75, 15), while leaflet readers’ mean score was 9.25 (IQR 8, 11). On the first viewing/reading at T1, there was no significant difference (0.5 (95% CI − 0.7, 1.7)) in the mean knowledge scores of participants considering the Onco*type* DX information (mean = 11.55) and those considering the Prosigna information (mean = 11.07). (Table 2 shows summary knowledge change score statistics recorded for individual participants dependent on whether they viewed the information films or leaflets first, and overall.

**Table 2:** Summary knowledge score statistics relating to changes recorded for individual participants dependent on whether they viewed the information film first or the leaflet, and overall

Group ID	*n*	Min	Max	IQR	SD	Mean	median
Onco*type* DX leaflet then film	30	1	9	3.75	2.58	5.33	6
Onco*type* DX film then leaflet	30	− 10	5	4.75	3.16	− 1.97	− 2
Prosigna leaflet then film	30	3	13	2.00	2.13	6.83	7
Prosigna film then leaflet	30	− 5	7	3.75	2.75	− 1.13	− 1
Onco*type* DX	60	− 10	9	8	4.66	1.68	1
Prosigna	60	− 5	13	8	4.70	2.85	3

### Secondary outcomes

Knowledge scores were always higher following film viewing than those after reading leaflets; mean scores for films were 13.37 at T1 and 15.33 at T2 whereas mean scores for leaflets were 9.25 at T1 and 11.82 at T2.
  If participants read a leaflet first and then viewed a film, mean knowledge scores at T2 increased by 6.08 points from 9.25 to 15.33 (*p* < 0.0001; 95% CI 5.44, 6.7). When the film was viewed first then the leaflet read, mean knowledge scores at T2 decreased by 1.55 points from 13.37 to 11.82 (*p* < 0.001; 95% CI − 2.32, − 0.78).
  Table 3 shows that following viewing of the films, T2 knowledge scores of all 120 participants improved whereas after reading the leaflets at T2, the knowledge of 8/60(13.3%) participants stayed the same, 16(26.7%) improved and 36(60%) declined.

**Table 3:** Participants (*N*%) whose knowledge scores stayed the same, improved or declined at T2 compared to T1

	All	Leaflet/film	Film/leaflet
	*N* (%)	*N* (%)	*N* (%)
Same	8 (6.7)	0	8 (13.3)
Improved	76 (63.3)	60 (100)	16 (26.7)
Declined	36 (30)	0	36 (60)
Total	120	60	60
There were significant differences in participants’ perceptions of the information contained in the different modalities: films were rated as follows: containing the ‘right amount’ of information (112/120 vs. 74/120), perceived as ‘very understandable’ (82/120 vs. 21/120) and ‘very well presented’ (113/120 vs. 59/120) compared to the leaflets (all differences significant at *p* < 0.0001).
Overall, irrespective as to whether or not its content referred to Onco*type* DX or Prosigna testing, there was a clear preference for the films (88 /120, 73.3%, 95% CI (0.643, 0.808). Only 28/120 (23.3%) favoured the leaflets and 4/120 (3.3%) expressed no preference.
(4) Participants gave numerous positive and negative reasons for their preferences and why different modalities had contributed more to their ease of understanding. Primary reasons for film preferences included its clarity, visual impact and emotional engagement:

“….film was really clear, concise and well-paced, allowed me to really listen. Not lots of medical terminology. More ordered than the leaflet, with a start, middle and end. Also, summary was excellent. It was easier for me to listen to someone telling me information than having to read it and at same time process it myself.”

“For me the film felt more reassuring, made it very clear that not everyone will need extra treatment and it’s important to avoid chemotherapy side effects if it won’t help reduce the risk of the cancer coming back, it really helped me understand that message when before I would have assumed having extra treatment would always be more helpful.”

“The film was very clear, I understood reasons why the test should be done, it was also good to know that another operation wasn’t necessary to have the test. Information was given at a good pace to take it in and I really liked that it was summarised at the end. For me the film covered everything in a simple way, had all the facts and the summary was so helpful for me - very much clearer than the leaflet.”

Interestingly some participants focussed on the fact that women with newly diagnosed breast cancer might be making decisions at a time of great anxiety and that the films were reassuring and comforting.

“If I were having to make a decision about taking this test without doubt the film is extremely helpful. It was well edited, easy to listen to, importantly I really liked the narrator, speech was slower than usual but I realised this was spot-on, it felt nice and clear and gentle, gave time for processing the information being delivered.” I liked the accompanying visual pieces, they re-iterated what was being said, made it engaging, it was nice to see the consultations happening, demonstrating the partnership of the decision-making process, it gave a sense of the supportive situation really well, which if you are in an anxious state is so important.”

The visual material used, especially the authenticity of vignettes of doctors with patients, appealed to several participants and aided their sense of support.

I preferred the film because the leaflet was boring, cumbersome, not my style, too wordy. In contrast the film had a good structure, a start, middle and end. It had much more of a warm & fuzzy feel about it, very human and sensitive. I liked that there was a wide range of women portrayed in it, the clips were sensible and not sensational, very caring, very well put together. It felt very real—real women, it felt supportive.

The film feels more relaxed and less intimidating. The leaflet felt more formal and clinical. The visuals in the film felt more authentic than the pictures in the leaflet, not having authenticity makes me switch off.

One participant also commented on the way in which much of our information now arrives in a digital format.

…. I really liked the film, because it was accessible, only 8 min, it was much less turgid. It is much more engaging, engages more of your senses, for me more information goes in. The film feels a more modern way to convey information, we expect information to be available this way on our phones and devices.

Negative reactions to the films were uncommon but included difficulties in finding information to replay easily, a dislike of the music and a general aversion to filmed material.

Those who preferred a leaflet frequently cited its easier accessibility and utility -

I could go back and forth more easily rather than going through the whole film again might be an age thing.

I like to have information in my hand to refer to, means I don’t have to tackle the computer each time. Using technology adds anxiety for me

I find a leaflet in your hand lets you re-read bits over and you can easily skip around or pass over bits. The other reason is that I would take it with me for my meeting with the doctor and use it to refer to bits I wanted to discuss.

Some mentioned that they would always like to have a leaflet even if they had enjoyed and understood the film.

“I’m a lawyer so I just like to read things!”

There were many negative comments made about the leaflets including uncertainty as to whom they were aimed at:

The leaflet was too technical and felt like sales or promotional material.

I did find it off-putting that the test was trade-marked in the leaflet, it very much felt that the test was being marketed. I also found the case studies with smiling faces inappropriate- too cheesy-not serious enough.

Other participants became confused by what they regarded as superfluous information.

…too much information was included, it seemed to have some material that was unnecessary, it distracted me and I wanted to skip past it, eg there was a whole page on what happens with the sample, details of what happens in the laboratory.

For me what was important to know was that I didn’t need an extra procedure and that the result took two weeks. That could be said in one sentence. I would be struggling to make a decision when clouded with so much information.

## Discussion

Communicating complex test results that might influence important decision-making is undoubtedly difficult. ASCO guidelines [16] mentioned the need to educate patients more about GEP testing, and how recurrence risk results affect adjuvant therapy planning. Although guidelines stressed the importance of effective communication, no specific studies were cited that addressed this area. During verbal interactions proficient communicators can in principle, change the terminology, content and presentation of information according to the perceived literacy and numeracy of their patient. Information leaflets, even some written with input from informed patients, may fail to promote the health literacy desired for good understanding, as they have a fixed content or may have been written at a level too high for the reading skills of the general population.

Our film format alone was more effective at aiding participants’ knowledge about GEP testing than the written material alone irrespective as to whether or not they described Onco*type*DX or Prosigna. A majority of participants found the film engaging, reassuring and supportive. They especially valued the tone and pace of the voice-over and the visual content with real people portrayed. A review concluded that showing people in filmed materials rather than mere didactic delivery of information is important [17].

The order effect of both viewing and reading the film and leaflet was interesting. Seeing a film first enabled slightly better understanding subsequently of the written information than that achieved after reading the leaflet alone. Likewise reading a leaflet first then viewing the film appeared to increase mean knowledge more than that after seeing the film alone. However, overall knowledge was still significantly higher following viewing of the films irrespective of the order or as to which GEP test was being explained. Although some participants commented on the usefulness of perhaps having both film and leaflet, others felt confused by the superfluous information in leaflets which led some to poorer recall and understanding than with film alone.

Although our study was conducted with participants without breast cancer due to the SARS-COV pandemic hampering our ability to access patients in clinic, the group were aged 45–75 and from varied socio-educational backgrounds typical of a breast cancer population. Discussions about GEP testing are conducted against a backdrop of fear and anxiety with women newly diagnosed and still coming to terms with their diagnosis. Helping their understanding in a sensitive manner is therefore vital and many participants commented that if they had been in the situation of a patient, then the leaflet would have increased uncertainty and anxiety whereas the film felt reassuring.

Well-written accessible patient information leaflets that do not overestimate health literacy and numeracy of the lay population can complement HCPs’ discussions about complex issues. Some patients may always prefer to have written information but we saw no evidence that this had any association with socio-educational grouping. We did see enhanced knowledge following viewing of the information films and a clear preference for this modality. Watching a film might well ease the discussions between HCPs and their breast cancer patients and lead to more informed shared decision-making about chemotherapy. We will be testing these hypotheses in clinics in a randomised study comparing standard information (verbal and/or with information leaflets) usually given by clinicians plus or minus the films in the next phase of the IMPARTER study.
